# Association between TyG index and risk of carotid atherosclerosis in NAFLD patients: a retrospective cohort study

**DOI:** 10.3389/fendo.2024.1448359

**Published:** 2024-08-20

**Authors:** Wei Huang, Hua Wang, Zhimei Shen, Xu Wang, Xiaosong Yu

**Affiliations:** ^1^ Health Management Center, Northern Jiangsu People’s Hospital Affiliated to Yangzhou University, Yangzhou, China; ^2^ Department of Trauma Surgery, Northern Jiangsu People’s Hospital Affiliated to Yangzhou University, Yangzhou, China; ^3^ Department of General Medicine, The First Affiliated Hospital of China Medical University, Shenyang, China

**Keywords:** triglyceride-glucose index, NAFLD, cohort study, carotid atherosclerosis, insulin resistance

## Abstract

**Background:**

The TyG index, or triglyceride-glucose index, is primarily used as a marker to assess insulin resistance and metabolic health. It increases mortality risk in patients with NAFLD, atherosclerosis, ischemic stroke, or heart failure. However, its association with Carotid Atherosclerosis (CAS) risk in NAFLD patients remains uncertain.

**Methods:**

This retrospective cohort study enrolled 739 individuals who participated comprehensive health evaluations at a large public hospital in Yangzhou, China, between January 2021 and December 2023. Among them, 436 were men and 303 were women, and their mean (SD) age was 51.53 ± 11.46 years. The individuals were categorized into three tertiles (Q1, Q2, and Q3), according to the baseline TyG index. Our investigation focused on exploring the correlativity between the TyG and the occurrence of CAS utilizing Cox regression and RCS analyses.

**Results:**

During a 3-year follow-up period, 199 patients developed CAS (cumulative incidence rate: 26.93%). A statistical model, adjusted for age, gender, BMI, and other confounders indicated that the HR (95%CI) values for CAS risk in the Q2 and Q3 groups were 3.11(1.87-5.17) and 4.51(2.69-7.56), respectively, with P-values <0.001 for both groups. A sensitivity analysis confirmed these results. Kaplan–Meier survival analysis revealed that CAS risk varied across the groups (*P* non-linear < 0.05).

**Conclusion:**

In individuals diagnosed as NAFLD, the possibility for CAS escalates with the elevation of the TyG value. Therefore, the TyG index is an effective marker for assessing the risk of CAS within this demographic. Large-sample prospective studies are needed to confirm this conclusion in the future.

## Introduction

Nonalcoholic fatty liver disease (NAFLD) manifests with a staggering global prevalence, estimated at 25% (1). Notably, it stands as the prevailing liver ailment within the people of China (2). Compared with individuals without NAFLD, those with this disease exhibit an elevated rate of mortality (3), with cerebrovascular diseases being the leading factor of death rather than the liver disease itself (1, 4). Although many researchers have explored the correlation between NAFLD and cardiovascular diseases (including ischemic heart disease, cardiac arrhythmias, hypertension) progression (5, 6), the potential risk of carotid atherosclerosis (CAS) has been overlooked. Evidence suggests that >30% of all patients with NAFLD develop CAS (7), a major cause of ischemic stroke, which carries high risks of disability and mortality. Identifying predictive factors for CAS in patients with NAFLD is crucial for reducing stroke incidence.

Insulin resistance (IR) is instrumental in the progression of NAFLD and worsens as the disease progresses (8, 9). It leads to endothelial dysfunction and oxidative stress, promoting atherosclerosis (10). The gold standard for diagnosing it is the hyperinsulinemic-euglycemic clamp (11, 12); however, the complexity of this technique limits its clinical utility and widespread application. The Homeostasis Model Assessment is another common indicator of IR (13), but its variability across populations complicates the determination of an optimal threshold (14). Moreover, this approach is unsuitable for patients receiving insulin therapy. The TyG index emerges as a novel metric for appraising IR within clinical settings (15, 16). In a state of insulin resistance, adipose tissue becomes less sensitive to insulin, leading to increased lipolysis and the release of free fatty acids into the bloodstream, which in turn leads to increased triglyceride synthesis in the liver. In addition, the presence of insulin resistance impairs the ability of insulin to promote glucose uptake and utilization, leading to elevated blood glucose levels. The TyG index indirectly reflects the degree of metabolic abnormalities in the body under insulin resistance by comprehensively assessing two indicators: fasting triglycerides and fasting blood glucose. Substantial evidence emphasizes a notable association between the TyG index and IR using the HIEC technique (17). Because of its reliability and ease of calculation, the TyG index emerges as especially well-suited for utilization resource-limited community hospitals (18). Additionally, research has shown that TyG-WC (waist circumference) can also reflect the state of IR (19).

This study aimed to investigate the correlation between the TyG index and susceptibility to CAS in NAFLD patients. The main goal was to enhance strategies for preventing CAS within this population.

## Materials and methods

### Study population

**Figure 1:**
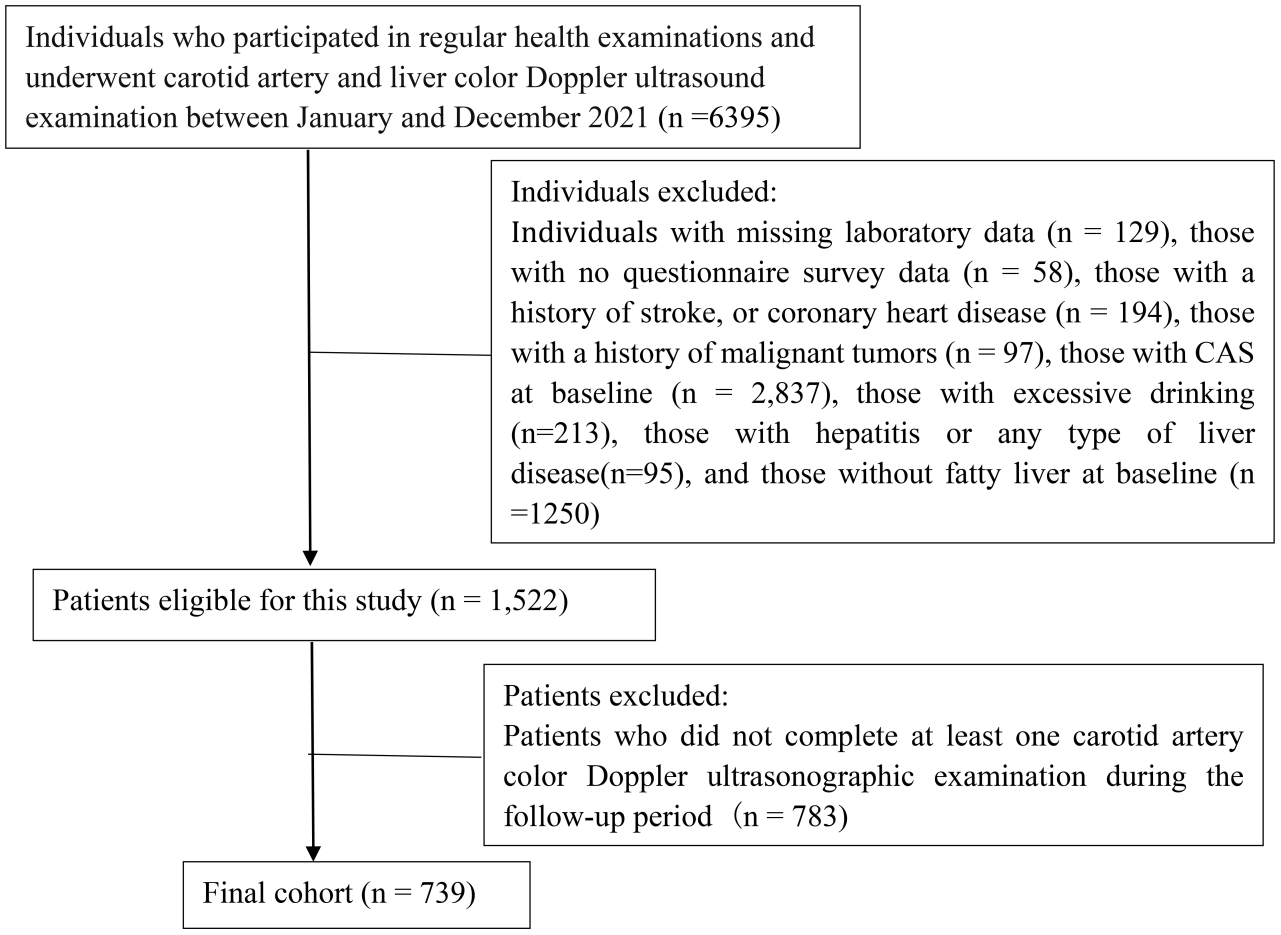
The flowchart depicting the process of patient selection.

Our study included 739 individuals who had undergone routine health examinations at Northern Jiangsu People’s Hospital Affiliated to Yangzhou University from January 2021 to December 2023. The study population comprised 436 men and 303 women. The patients’ mean (SD) age was 51.53 ± 11.46 years. The inclusion criteria were outlined as follows: undergoing regular health examinations at our hospital at least once; completing baseline carotid artery color Doppler ultrasonography, liver color Doppler ultrasonography, and laboratory examinations; having NAFLD at baseline; and not having CAS at baseline. The exclusion criteria encompassed individuals with a history of stroke, coronary heart disease, or malignant neoplasms; those engaging in excessive drinking; individuals with hepatitis or any type of liver disease; participants lacking questionnaire survey data and laboratory test results; and subjects who did not complete at least one carotid artery color Doppler ultrasonographic examination during the follow-up period. A flowchart depicting the enrollment of patients is presented **Figure 1**. Our study received approval from the Ethics Committee of Northern Jiangsu People’s Hospital Affiliated to Yangzhou University (approval number: 2024ky098). Adhered to the ethical precepts delineated in the Declaration of Helsinki as well as other relevant regulations. Owing to the retrospective nature of the investigation, our institutional review board waived the need for informed consent.

### Data collection

We collected data on basic characteristics such as the patients’ age, sex, smoking status (yes/no), previous medical history, medication use, and anthropometric measurements (height and weight). In the morning, 5 mL of fasting venous blood samples were collected. Laboratory measurements comprised fasting blood glucose and fasting blood lipids. Blood pressure was measured on the right brachial artery by using an electronic sphygmomanometer. The patients rested in a seated position with their feet naturally flat on the ground for at least 5 min.

Carotid artery color Doppler ultrasonography was performed with the patient in the supine position. Experienced ultrasound physicians conducted the examinations, commencing with a transverse scan from the neck root toward the head. They particularly evaluated the proximal, middle, and distal segments of the carotid artery, including the carotid bifurcation, and both the internal and external carotid arteries. This was followed by a longitudinal scan along the long axis of the carotid artery; finally, the blood flow velocity of the arterial lumen was determined through color Doppler flow imaging.

### Definitions and diagnostic criteria

NAFLD was diagnosed on the basis of the following criteria: the presence of hepatic steatosis, the absence of excessive alcohol consumption (alcohol consumption <20 g/day in women and<30 g/day in men), and the absence of other liver diseases (20). Criteria for ultrasonic diagnosis of fatty liver (two or more of the subsequent conditions must be met): enhanced echogenicity in the anterior segment of the liver, exhibiting a ‘bright liver’ characteristic; attenuation of far-field echoes; obscured visualization of intrahepatic duct structures (21).

The criteria for ultrasonic diagnosis of carotid atherosclerosis are intima-media thickness (IMT) > 1.0mm, or the detection of plaque formation. Carotid plaques were identified by IMT ≥1.5 mm, or IMT value that exceeded the surrounding area by ≥50% (22, 23).

Hypertension was defined as systolic blood pressure ≥18.7Kpa (140 mmHg), or diastolic blood pressure ≥12.0Kpa (90 mmHg). Patients who have been definitively diagnosed and are undergoing antihypertensive treatment, even if their blood pressure is below 140/90 mmHg, are still considered to have hypertension (24). Diabetes was defined by FPG ≥7.0 mmol/L, diagnosed diabetes, a hemoglobin A1c level of ≥6.5%, or existing use of oral or subcutaneous antidiabetic medications (25). TyG index =ln [TG × FBG/2]. TG and FBG were presented in terms of milligrams per deciliter.

### Statistical analysis

We performed statistical analyses utilizing SPSS (version 25.0), R (version 4.1.3), and EmpowerStats software. Normally distributed data are typically presented in terms of M ± SD, whereas nonnormally distributed data are presented in terms of median (25th percentile and 75th percentile) values. Between-group comparisons were conducted utilizing either the *t*-test or the Mann–Whitney U test., and multigroup comparisons were performed using ANOVA or Kruskal–Wallis test. Categorical data were analyzed using χ² test.

Kaplan–Meier curves, plotted based on TyG index tertiles, depicted the cumulative incidence rates of CAS events. We utilized the log-rank test for between-group comparisons. Cox proportional hazards regression was performed to calculate HR and 95% CI values for the correlation between the TyG index and CAS incidence. When Omnibus test P < 0.05, it indicated the constructed COX regression model was significant. The adjustment of covariates is determined by the results of inter-group comparisons, common clinical risk factors and multicollinearity tests. When the variance inflation factor (VIF) was greater than 5, it indicated the presence of multicollinearity, which necessitates its exclusion. Additionally, both the TyG ×BMI and TyG index were indicators used to assess insulin resistance, and TyG ×BMI was the product of TyG and BMI. In the multivariate COX regression, TyG×BMI was not included. Three statistical models were used for data analysis. Model 1 had no control variables. Model 2 had controls for age, sex, smoking, history of hypertension and diabetes. Model 3 had controls for sex, age, smoking, history of hypertension, diabetes, SBP, anti-hypertension medication, antidiabetic agents, lipid-lowering medication, FBG, TC. TG was screened out because of covariance. A four-knot RCS analysis was conducted to examine the nonlinear correlation between the TyG index and the incidence of CAS in NAFLD patients. Using the 5th percentile as a benchmark, knots were established at 5th, 35th, 65th, and 95th percentiles of TyG measures.

Subgroup analyses were performed to test interactions among age groups (<50 or ≥50 years), sex groups, and BMI-based groups (<24 or ≥24 kg/m²). P for interaction >0.05 indicated that there was no significant difference between groups. Sensitivity analyses (using TyG quartile grouping and excluding subjects with a follow-up time of less than 9 months) were conducted to confirm the consistency of the findings. Missing values, including 31 cases of blood pressure values and 23 cases of height and weight values, were addressed using multiple imputation methods.

## Results

### The demographic characteristics and clinical data comparison of the study population

This study included 739 patients (mean age: 51.53 ± 11.46 years). The follow-up duration was 708 (568, 730) days (minimum: 200 days; maximum: 1045 days). The mean ± standard deviation of TyG was 7.52 ± 0.64. The patients were stratified into three groups by TyG index tertiles: Q1 (TyG index value ≤ 7.21), Q2 (TyG index value: 7.21–7.70) and Q3 (TyG index value > 7.70) groups. No significant between-group difference was noted in age, sex, smoking, BMI, anti-hypertension medication, antidiabetic agents, lipid-lowering medication or follow-up duration (*P* = 0.178, 0.418, 0.076, 0.429, 0.032, 0.160, 0.788 and 0.816, respectively). Compared to Group Q1, Groups Q2 and Q3 exhibited higher prevalence rates of hypertension and diabetes, as well as higher levels of FBG, TG, TC, LDL-C, and metabolic indexes (including TyG×BMI and TyG). However, their HDL-C levels were lower (**Table 1**).

**Table 1:** General characteristics of the study population (n = 739).

TyG group	N	Q1 ≤7.21	Q2 7.21-7.70	Q3 >7.70	*P*-value
Sample Size (n)	739	244	248	247	
Age(years)	51.53 ± 11.46	50.69 ± 11.79	52.57 ± 11.48	51.31 ± 11.06	0.178
Gender(n, %)					0.418
male	436 (59.00%)	139 (56.97%)	143 (57.66%)	154 (62.35%)	
female	303 (41.00%)	105 (43.03%)	105 (42.34%)	93 (37.65%)	
Smoking(n, %)	107 (14.48%)	30 (12.30%)	31 (12.50%)	46 (18.62%)	0.076
Hypertension(n, %)					**0.009**
yes	160 (21.65%)	41 (16.80%)	50 (20.16%)	69 (27.94%)	
no	579 (78.35%)	203 (83.20%)	198 (79.84%)	178 (72.06%)	
Diabetes(n, %)					**< 0.001**
yes	105 (14.21%)	4 (1.64%)	21 (8.47%)	80 (32.39%)	
no	634 (85.79%)	240 (98.36%)	227 (91.53%)	167 (67.61%)	
Systolic Blood Pressure (SBP, mmHg)	132.47 ± 17.74	128.36 ± 17.46	132.39 ± 16.06	136.61 ± 18.71	**< 0.001**
Diastolic Blood Pressure (DBP, mmHg)	79.20 ± 10.85	77.62 ± 10.89	78.87 ± 10.19	81.09 ± 11.21	**0.002**
Body Mass Index(BMI, kg/m^2^)	26.04 ± 2.78	25.97 ± 2.66	26.22 ± 2.85	25.92 ± 2.83	0.429
Fasting Blood Glucose(FBG, mmol/L)	5.32 (4.87-6.01)	5.01 (4.63-5.36)	5.30 (4.87-5.87)	5.87 (5.25-7.59)	**< 0.001**
Triglycerides(TG, mmol/L)	1.85 (1.40-2.77)	1.27 (1.10-1.45)	1.87 (1.69-2.16)	3.32 (2.71-4.64)	**< 0.001**
High-Density Lipoprotein Cholesterol (HDL-C, mmol/L)	1.13 ± 0.26	1.23 ± 0.26	1.15 ± 0.23	1.01 ± 0.23	**< 0.001**
Low-Density Lipoprotein Cholesterol (LDL-C, mmol/L)	3.02 ± 0.82	3.03 ± 0.71	3.17 ± 0.80	2.85 ± 0.91	**< 0.001**
Total Cholesterol (TC, mmol/L)	4.87 ± 0.92	4.61 ± 0.81	4.87 ± 0.89	5.13 ± 0.98	**< 0.001**
Anti-hypertension medication (n, %)	62 (8.39%)	15 (6.15%)	17 (6.85%)	30 (12.15%)	**0.032**
Antidiabetic agents (n, %)	37 (5.01%)	7 (2.87%)	16 (6.45%)	14 (5.67%)	0.160
Lipid-lowering medication (n, %)	12 (1.62%)	3 (1.23%)	5 (2.02%)	4 (1.62%)	0.788
Follow-up Time (days)	708.00 (568.00-730.00)	707.00 (566.00-729.25)	708.50 (533.25-734.00)	708.00 (614.50-729.00)	0.816
TyG ×BMI	195.85 ± 26.62	179.25 ± 19.81	194.58 ± 21.35	213.52 ± 26.31	**< 0.001**
TyG	7.52± 0.64	6.90 ± 0.24	7.42 ± 0.14	8.24 ± 0.48	**< 0.001**

BMI, body mass index; TyG, triglycerides- glucose index; the bold values, P<0.05.

### Incidence of CAS in patients With NAFLD

**Figure 2:**
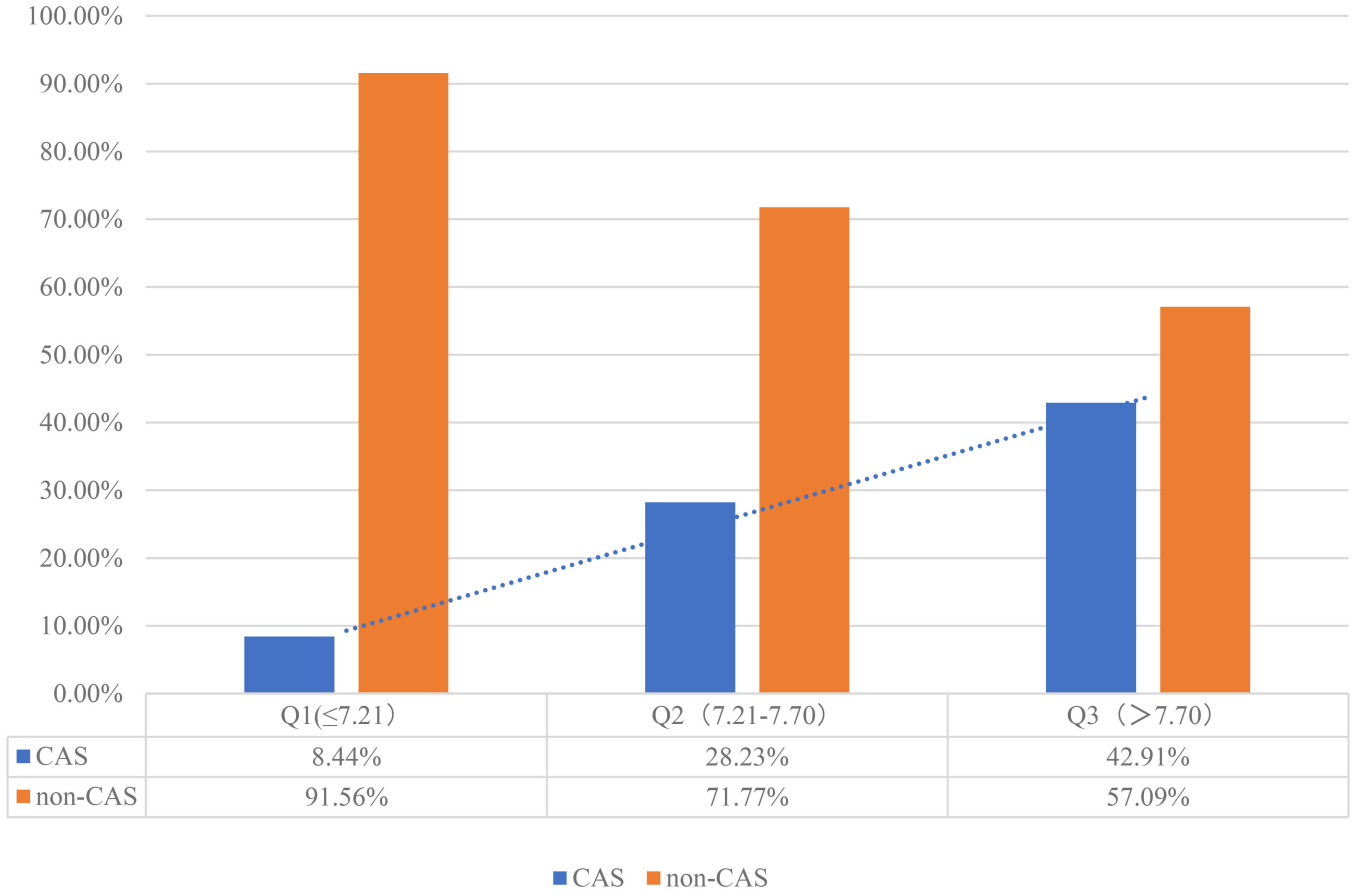
The incidence of CAS in NAFLD patients across different TyG groups.

Of the 739 patients with NAFLD who were monitored for 3 years, 199 developed CAS (cumulative incidence rate: 26.93%). The annual incidence rate was 1,238/10,000 person-years or 12.38% person-years (95% CI: 10.34-14.70%). The incidence rate of CAS was higher in the Q2 and Q3 groups than in the Q1 group (8.44% vs. 23.23% [*P* < 0.001] and 8.44% vs. 42.91% [*P* < 0.001], **Figure 2**).

### Clinical and biochemical characteristics of individuals with or without CAS

Our individuals with NAFLD were sorted into two groups based on the occurrence of CAS throughout the follow-up period: those with CAS and those without CAS. Regrading general characteristics, no significant between-group difference was noted in sex distribution (*P* = 0.212). Patients with CAS had higher SBP compared to those without CAS (*P* < 0.001). There are differences in the use of antihypertensive drugs and hypoglycemic drugs between the two groups, and the difference is statistically significant (P = 0.005, 0.007, respectively). Furthermore, the values of IR indicators, such as TyG×BMI value, TyG index, were notably elevated in patients with CAS compared to those without (*P <* 0.05), and the difference between groups in TyG index was greater, with higher levels of TyG in CAS patients. There were no notable differences observed in HDL-C, LDL-C, or lipid-lowering medication between the groups (*P* > 0.05; **Table 2**).

**Table 2:** Clinical and biochemical characteristics of patients with or without CAS.

	NAFLD	NAFLD-CAS	Standardize diff.	*P*-value
Cases(n)	540	199		
Age(years)	50.46 ± 11.38	54.43 ± 11.21	0.35 (0.19, 0.51)	**< 0.001**
Gender(n, %)			0.10 (-0.06, 0.27)	0.212
male	326 (60.37%)	110 (55.28%)		
female	214 (39.63%)	89 (44.72%)		
Smoking(n, %)	79 (14.63%)	28 (14.07%)	0.02 (-0.15, 0.18)	0.848
Hypertension(n, %)			0.20(0.04,0.36)	**0.005**
yes	103 (19.07%)	57 (28.64%)		
no	423 (75.13%)	137 (65.87%)		
Diabetes(n, %)			0.51 (0.34, 0.67)	**< 0.001**
yes	49 (9.07%)	56 (28.14%)		
no	491 (90.93%)	143 (71.86%)		
Systolic Blood Pressure (SBP, mmHg)	130.64 ± 17.20	137.44 ± 18.25	0.38 (0.22, 0.55)	**< 0.001**
Diastolic Blood Pressure (DBP, mmHg)	78.86 ± 10.74	80.11 ± 11.12	0.11 (-0.05, 0.28)	0.166
Body Mass Index(BMI, kg/m^2^)	26.04 ± 2.80	26.04 ± 2.73	0.00 (-0.16, 0.16)	1.000
Fasting Blood Glucose(FBG, mmol/L)	5.19 (4.80, 5.75)	5.72 (5.19, 7.09)	0.51 (0.34, 0.67)	**< 0.001**
Triglycerides(TG, mmol/L)	1.72 (1.32, 2.48)	2.23 (1.79, 3.16)	0.28 (0.12, 0.44)	**< 0.001**
High-Density Lipoprotein Cholesterol (HDL-C, mmol/L)	1.13 ± 0.26	1.13 ± 0.25	0.01 (-0.15, 0.17)	0.884
Low-Density Lipoprotein Cholesterol (LDL-C, mmol/L)	2.99 ± 0.79	3.10 ± 0.88	0.14 (-0.02, 0.30)	0.082
Total Cholesterol (TC, mmol/L)	4.78 ± 0.90	5.10 ± 0.94	0.35 (0.18, 0.51)	**<0.001**
Anti-hypertension medication (n, %)	103 (19.07%)	57 (28.64%)	0.23 (0.06, 0.39)	**0.005**
Antidiabetic agents (n, %)	20 (3.70%)	17 (8.54%)	0.20 (0.04, 0.37)	**0.007**
Lipid-lowering medication (n, %)	10 (1.85%)	2 (1.01%)	0.07 (-0.09, 0.23)	0.419
TyG ×BMI	193.09 ± 26.11	203.32 ± 26.61	0.39 (0.22, 0.55)	**<0.001**
TyG	7.42 ± 0.62	7.81 ± 0.59	0.65 (0.48, 0.81)	**<0.001**

BMI, body mass index; TyG, triglycerides- glucose index; the bold values, P<0.05.

### Association between the TyG index and CAS incidence among individuals with NAFLD

**Figure 3:**
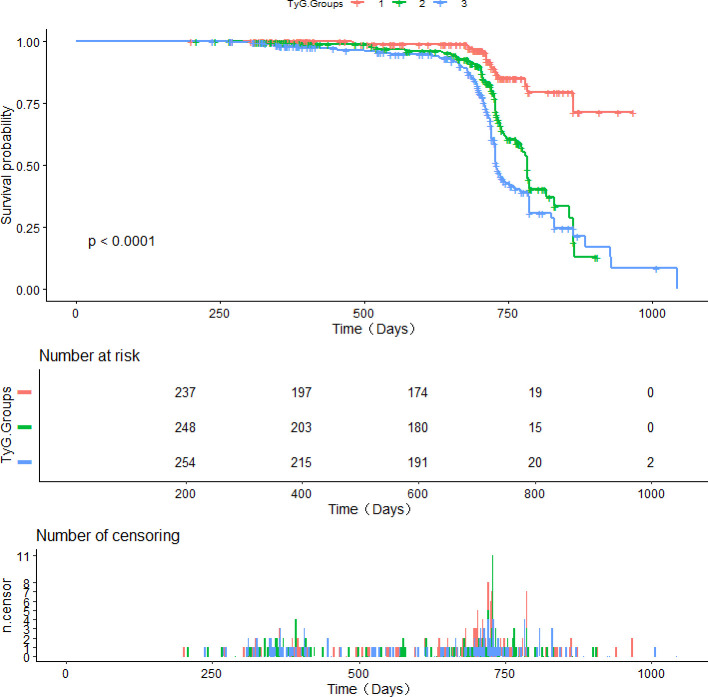
Survival curves derived from Kaplan-Meier analysis for the incidence of CAS in patients with NAFLD.

Kaplan–Meier curves for CAS incidence indicated that a heightened baseline TyG was linked to an elevated likelihood of CAS (Q1 vs. Q2 and Q1 vs. Q3, log-rank *P* < 0.001; Q1 vs. Q3, log-rank *P* < 0.001; **Figure 3**).

**Figure 4:**
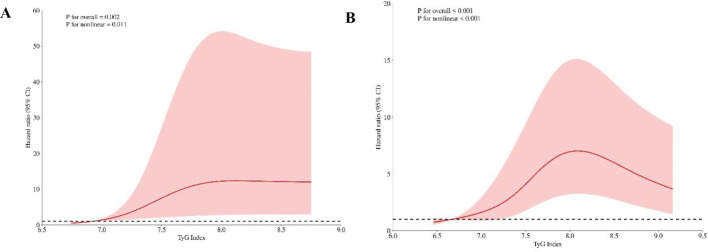
RCS plots depicting the risk ratio of the TyG in different BMI groups. **(A)** BMI<24kg/m^2^. **(B)** BMI≥24kg/m^2^. **(A)** shows TyG index 6.71 and **(B)** shows TyG index 6.65 as the references. The vertical axis illustrates the hazard ratio (HR), while the red shaded region depicts the 95% confidence interval (CI) value. The horizontal dashed line signifies an HR of 1.

Cox regression was performed to investigate the relationship between the TyG index and CAS incidence among individuals diagnosed with NAFLD (**Table 3**). Omnibus test P were <0.001 in all constructed COX regression models. The TyG value, when treated as a continuous variable, was found to be linked with the incidence of CAS. This association remained significant even after covariate adjustment (adjusted HR 1.39; 95% CI: 1.10 - 1.76; *P* = 0.006). When the TyG index was assigned as a qualitative variable, with Q1 as the reference group, Model 1 revealed that the HR for CAS incidence in the Q3 group was 5.23 (95% CI: 3.28 - 8.36; *P* < 0.001). Model 3 showed a positive relationship between the TyG index and the occurrence of CAS in the Q2 and Q3 groups.; notably, this association was the strongest in the highest tertile group of TyG group (HR: 4.51; 95% CI: 2.69 - 7.56; *P* < 0.001). The univariate Cox regression analysis showed that there was a correlation between TyG × BMI and CAS, but the correlation was relatively low (HR = 1.009, 95% CI: 1.004 - 1.013, *P* < 0.001). Furthermore, the RCS analysis unveiled a dose–response correlation between the TyG index and CAS incidence in individuals diagnosed with NAFLD. The nonlinear correlation between TyG and CAS risk was consistent when BMI was divided into whether ≥ 24 kg/m² or not (*P* non-linear < 0.05; **Figure 4**).

**Table 3:** Association between the baseline TyG and CAS incidence in individuals with NAFLD.

Models	Model1 HR (95% CI)	*P*-value	Model2 HR (95% CI)	*P*-value	Model3 HR (95% CI)	*P*-value
Continuous TyG	1.60(1.35-1.90)	***P* < 0.001**	1.50(1.22-1.83)	***P* < 0.001**	1.39(1.10-1.76)	***P* = 0.006**
Q1 ≤7.21	1.00		1.00		1.00	
Q2 7.21-7.70	3.48(2.13-5.67)	***P* < 0.001**	3.34(2.04-5.47)	***P* < 0.001**	3.11(1.87-5.17)	***P* < 0.001**
Q3 >7.70	5.23(3.28-8.36)	***P* < 0.001**	4.77(2.92-7.78)	***P* < 0.001**	4.51(2.69-7.56)	***P* < 0.001**

Model1: Crude model.

Model2: Adjusted for sex, age, smoking, history of hypertension, diabetes.

Model3: Adjusted for sex, age, smoking, history of hypertension, diabetes, SBP, anti-hypertension medication, antidiabetic agents, lipid-lowering medication, FBG, TC.

The bold values, P<0.05.

### Subgroup analysis

Further analyses consistently revealed a positive relationship between the TyG and CAS incidence across patients stratified by age, sex, BMI, or hypertension history (*P* for interaction = 0.397, 0.620, 0.101, and 0.686, respectively; **Table 4**). Notably, the TyG index exhibited superior predictive performance in patients with NAFLD without diabetes (HR: 1.82; 95% CI: 1.44–2.31) than diabetic patients (HR: 0.93; 95% CI: 0.65–1.32); the *P* value for interaction was 0.013.

**Table 4:** Forest plot depicting the risk ratio of CAS across the subgroups.

Subgroups	HR	95%CI		*P*-value	*P*-interaction
Sex			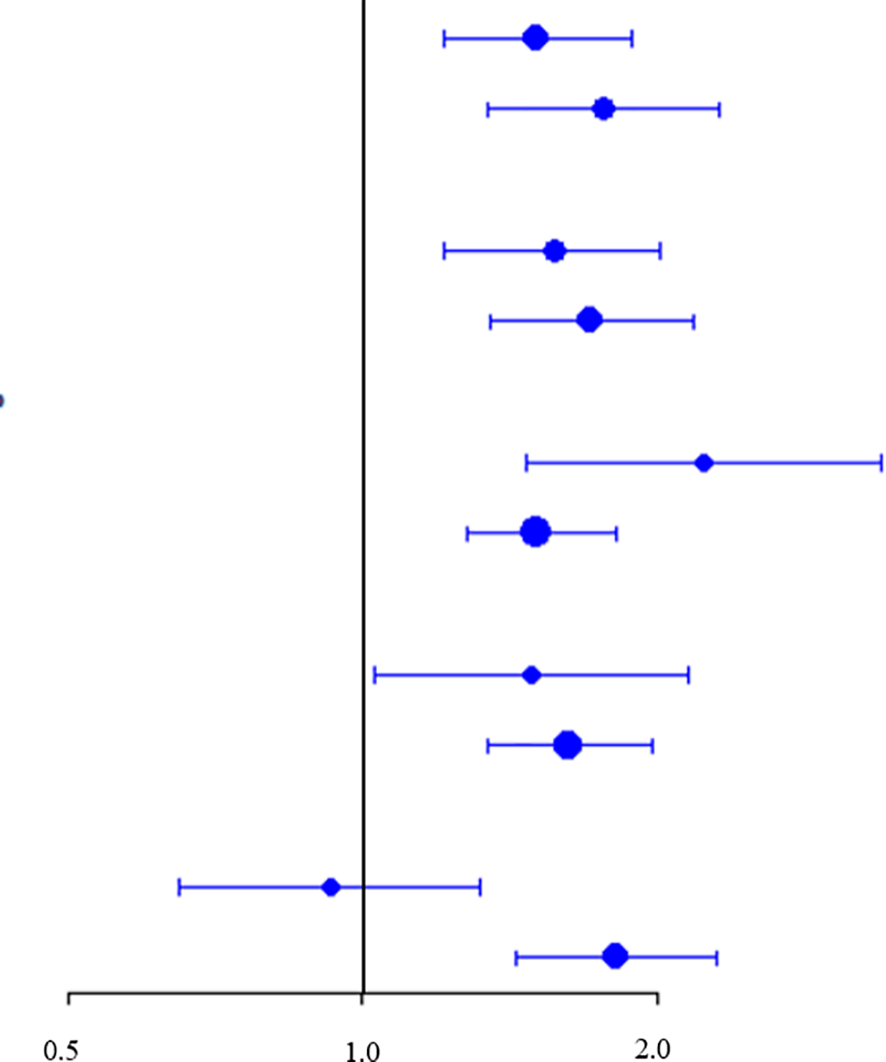		0.397
Male	1.51	(1.21, 1.89)		<0.001	
Female	1.76	(1.34, 2.32)		<0.001	
Age(years)					0.620
≤50	1.57	(1.21, 2.02)		<0.001	
>50	1.71	(1.35, 2.18)		<0.001	
BMI(kg/m2)					0.101
<24	2.23	(1.47, 3.39)		<0.001	
≥24	1.51	(1.28, 1.82)		<0.001	
Hypertension					0.686
Yes	1.49	(1.03, 2.16)		0.034	
No	1.62	(1.34, 1.98)		<0.001	
Diabetes					**0.013**
Yes	0.93	(0.65, 1.32)		0.669	
No	1.82	(1.44, 2.31)		<0.001	

The bold values, P<0.05.

### Results of sensitivity analysis

The TyG index remained significantly associated with CAS after patient stratification by TyG index tertiles (**Supplementary Table S1**). After adjusting for all covariates, the HR (95% CI) was 4.34 (2.44 - 7.69), *P* <0.001. Even after the exclusion of patients who developed CAS within the first 9 months of follow-up, no notable change was detected in the aforementioned association (**Supplementary Table S2**). After adjusting for all relevant covariates, the HR (95% CI) was found to be 4.55 (2.74 - 7.56), with a p-value <0.001.

## Discussion

This longitudinal cohort study unveiled a robust positive correlation between the TyG and the CAS incidence in NAFLD individuals. The correlation remained consistent even after covariate adjustment, indicating that a heightened TyG value indicates an elevated risk of CAS. Thus, a high TyG value appears to independently contribute to CAS in patients with NAFLD. This study might represent the initial exploration into unveiling the predictive capability of the TyG index concerning CAS risk within this demographic.

Previous studies have shown that there is an association between the TyG index and carotid atherosclerosis (such as carotid plaque formation, carotid stenosis, and arterial stiffness) (23, 26, 27). However, the relationship between TyG and CAS in specific populations remains unclear and requires further assessment, as the TyG index is largely determined by the state of glucose and lipid metabolism. Individuals with familial hypercholesterolemia (FH), TyG was positively correlated with the incidence of atherosclerotic cardiovascular disease (ASCVD)[OR = 1.74(95%CI: 1.15–2.63, p <0.05)] (13). In patients with symptomatic coronary artery disease, the incidence of CAS was significantly higher in those with the highest quartile of the TyG index compared to those with the lowest quartile [OR = 2.31 (95% CI 1.27, 4.20), P < 0.05] (27). Our study revealed that in patients with metabolic-specific conditions (NAFLD), each unit increase in the TyG index was associated with a 39% increased risk of CAS (95% CI: 1.10 - 1.76; P = 0.006). In a study involving 26,765 subjects with BMI less than 25, an increase in the TyG index was observed to be associated with an increased prevalence of CAS. However, after adjusting for confounding factors, the statistical association between them became insignificant (28). In the subgroup analysis of this study, no significant impact of varying BMI levels on the relationship between the TyG index and CAS was observed. The I-Lan Longitudinal Aging Study (ILAS) unveiled compelling evidence suggesting that the TyG index holds significance in forecasting atherosclerosis in patients without diabetes, particularly women, but not in those with diabetes (29). The findings of Anxin Wang (30) and Yawen Lu et al. (29) are consistent with our research results. In diabetic patients, no relationship was found between TyG and CAS, which may be related to the use of hypoglycemic medications. Although the documented correlation between the TyG index and NAFLD is well-established (31–33). the potential role of TyG in predicting the possibility of cervical vascular diseases in these patients has not yet been fully explored and confirmed (34, 35).

Changes in TyG index values, determined on the metabolic status of glucose and lipid, suggest the potential of this index in predicting CAS in individuals with specific metabolic characteristics, such as those with NAFLD (36–38). However, further research is necessary to comprehensively understand the biological mechanisms that connect the TyG with the emergence of CAS in individuals with NAFLD. Beyond its association with IR, the TyG index may be influenced by factors such as inflammation, oxidative stress, gut function, and gut microbial dysbiosis.

IR is a major driver of both NAFLD and CAS. An excessively high intrahepatic TG level, a characteristic of NAFLD, and the occurrence of *de novo* lipogenesis (DNL) within the liver promote hepatic steatosis in individuals afflicted by NAFLD (39). Insulin resistance improves the plasma levels of insulin and glucose, further promoting DNL. DNL may produce toxic metabolites, such as ceramides, which can exacerbate IR (40, 41). IR also significantly contributes to the onset of atherosclerosis (9, 10). Insulin signaling is essential for activating nitric oxide (NO), and IR inhibits NO production. NO is a key vasodilator and antiatherosclerotic agent. A reduction in NO level impairs the phosphoinositide 3-kinase/NO pathway. Furthermore, an IR-induced increase in insulin level can activate the mitogen-activated protein kinase pathway. The imbalance between these two pathways can lead to endothelial dysfunction (38, 39) (. Furthermore, IR upregulates the production of endothelin-1, thus promoting vasoconstriction and atherosclerosis progression (42). Elevated TyG index values in NAFLD patients may reflect underlying IR, which may contribute to the progression of CAS.

NAFLD is a systemic chronic inflammatory condition. Adipose tissue plays a momentous role in the chronic inflammatory reaction associated with NAFLD (43). This tissue is not merely a passive storage site for excess energy but actively participates in the metabolic and inflammatory processes that characterize NAFLD. An imbalance between adipokine and cytokine levels can explain this association (37, 44). This imbalance manifests as reductions in the levels of anti-inflammatory adipokines (e.g., adiponectin) and increases in those of proinflammatory cytokines, including interleukin (IL)-8, IL-6, IL-1β, interferon (IFN)-γ, and so on. Hyperplasia and dysfunction of adipose tissue, particularly visceral adipose tissue, downregulate the synthesis of anti-inflammatory adipokines, while concurrently upregulating the synthesis of proinflammatory cytokines. This proinflammatory milieu in NAFLD promotes atherosclerosis, as demonstrated by the Canakinumab Anti-Inflammatory Thrombosis Outcomes Study (45).Insulin resistance (IR) leads to a chronic, low-grade inflammatory state in adipose tissue and other metabolically active tissues such as the liver. These tissues release a multitude of inflammatory mediators, including cytokines, chemokines, and adhesion molecules. These mediators are capable of attracting and activating inflammatory cells, such as monocytes and macrophages, towards the vascular wall. Once infiltrated into the vascular wall, these inflammatory cells release additional inflammatory mediators and growth factors, which stimulate the proliferation, migration, and phenotypic transformation of vascular smooth muscle cells (VSMCs) (46). Furthermore, they promote the synthesis and deposition of extracellular matrix (ECM) components. Collectively, these changes contribute to the formation and progression of atherosclerotic plaques. The TyG index, as a comprehensive indicator of lipid and glucose metabolism, may simultaneously reflect these two aspects of change. By integrating information on triglyceride levels and fasting glucose, the TyG index offers a glimpse into the underlying metabolic disturbances that precede and accompany atherosclerosis, including those driven by IR, lipid dysregulation, and chronic inflammation.

Furthermore, the onset and progression of NAFLD are associated with microbial flora (43, 47). NAFLD often features gut dysbiosis characterized by an increased abundance of *Escherichia coli* and a reduced abundance of *Prevotella* sp. These gut microbes convert dietary choline or carnitine to trimethylamine, which the liver converts to trimethylamine N-oxide. The metabolized form alters calcium signaling in platelets, leading to increased platelet reactivity and thrombosis, thus promoting atherosclerosis (37, 48). Studies have shown a link between insulin resistance and gut microflora (49). The TyG index may also be influenced by the state of the gut microbiota.

In recent years, the TyG index has gained increasing popularity and recognition in clinical practice due to its remarkable cost-effectiveness. This index has significantly broadened its application beyond merely assessing cardiovascular diseases, now encompassing the prediction of acute kidney injury risk (50). Furthermore, the TyG index has been firmly established as a crucial factor in evaluating the incidence of chest pain (51), offering a novel perspective for stratified management of patients experiencing this condition. Notably, the index has also demonstrated potential in the field of mental health, exhibiting a significant correlation with the risk of depression onset, thereby providing a new biomarker reference for early screening and the formulation of preventive strategies for depression (52).

The strength of this study lies in its 3-year longitudinal cohort design, which gave it an advantage over cross-sectional studies in causal inference. Our findings indicate that the prognostic capacity of the TyG for carotid atherosclerosis (CAS) rate is more pronounced in patients with NAFLD who do not have diabetes compared to those who do. Furthermore, we performed covariate adjustment and risk stratification analyses across various subgroups.

This study is subject to certain limitations. Firstly, due to the study’s single-center, retrospective cohort design, our findings may be subject to selection bias. Secondly, we used ultrasonography instead of the gold standard liver biopsy for diagnosing NAFLD. While liver biopsy provides the most definitive assessment by directly examining liver tissue, it is an invasive procedure with associated risks and discomfort for patients. Ultrasonography offers a non-invasive, safe, and widely accessible alternative. Despite its limitations compared to liver biopsy, ultrasonography is still an effective diagnostic tool for NAFLD, with a high sensitivity and specificity (20). Thirdly, despite performing multivariate Cox regression as well as subgroup and sensitivity analyses, we could not fully exclude potential confounders, such as diet and exercise. Fourthly, the patients were not stratified by the severity of NAFLD. We are unable to determine whether the correlation between Tyg and CAS varies across different levels of NAFLD. Finally, the RCS analysis indicated that the HR for CAS incidence in patients with NAFLD exhibited an upward trend with increasing TyG index values, until a threshold was reached; thereafter, a downward trend was noted. However, it is important to note that we cannot definitively conclude that the prognostic capacity of the TyG for the risk of carotid atherosclerosis (CAS) diminishes after reaching a certain threshold. This drawback arises due to the relatively limited sample size of individuals with high TyG index values in our study. Currently, our research is confined solely to NAFLD patients and cannot be generalized to the general population. Further research with larger cohorts is essential to confirm and extend these findings, facilitating a broader comprehension of the prognostic capability of the TyG across diverse stages among NAFLD patients.

## Conclusion

We noted that patients with elevated TyG demonstrated a notably increased possibility of developing CAS among those diagnosed with NAFLD. This association underscores the TyG as a crucial indicator in assessing the risk of atherosclerotic complications. It can be utilized for the early prevention and intervention of CAS, thereby improving patient outcomes and overall cerebrovascular health.

## Data Availability

The original contributions presented in the study are included in the article/**Supplementary Material**. Further inquiries can be directed to the corresponding authors.
