# Supplementary material for: Association between TyG index and risk of carotid atherosclerosis in NAFLD patients: a retrospective cohort study
Source: Front Endocrinol (Lausanne). 2024 Aug 20;15:1448359. doi: 10.3389/fendo.2024.1448359 (PMC11368734; doi:10.3389/fendo.2024.1448359)
Supplement: Supplementary file 1 [file Table1.docx]

Supplementary Material

**Supplementary Table S1. Relationship between the baseline TyG index and CAS incidence in individuals with NAFLD.**

| Models | Model1 HR (95% CI) | *P*-value | Model2 HR (95% CI) | *P*-value | Model3 HR (95% CI) | *P*-value |
| --- | --- | --- | --- | --- | --- | --- |
| Q1  ≤7.09 | 1.00 |  | 1.00 |  | 1.00 |  |
| Q2  7.09-7.40 | 2.85（1.57-5.17） | **0.001** | 2.56（1.40-4.69） | **0.002** | 2.55（1.39-4.69） | **0.003** |
| Q3  7.40-7.87 | 4.97（2.85-8.68） | ***P* ＜ 0.001** | 4.64（2.63-8.18） | ***P* ＜0.001** | 4.34（2.44-7.69） | ***P* ＜ 0.001** |
| Q4  ＞7.87 | 5.61（3.24-9.73） | ***P* ＜ 0.001** | 4.90（2.76-8.71） | ***P* ＜0.001** | 4.60（2.54-8.33） | ***P* ＜ 0.001** |

Model1: Crude model;

Model2: Adjusted for sex, age, smoking, history of hypertension, diabetes;

Model3: Adjusted for sex, age, smoking, history of hypertension, diabetes, SBP, anti-hypertension medication, antidiabetic agents, lipid-lowering medication, FBG, TC.

**Supplementary Table S2. Correlation between the TyG index and CAS incidence in individuals with NAFLD, excluding those developing CAS within the first 9 months of follow-up.**

| Models | Model1 HR (95% CI) | *P*-value | Model2 HR (95% CI) | *P*-value | Model3 HR (95% CI) | *P*-value |
| --- | --- | --- | --- | --- | --- | --- |
| Q1  ≤7.22 | 1.00 |  | 1.00 |  | 1.00 |  |
| Q2  7.22-7.70 | 3.47（2.13-5.66） | ***P* ＜0.001** | 3.34（2.04-5.47） | ***P* ＜ 0.001** | 3.14（1.91-5.16） | ***P* ＜ 0.001** |
| Q3  ＞7.70 | 5.24（3.28-8.37） | ***P* ＜0.001** | 4.77（2.92-7.80） | ***P* ＜ 0.001** | 4.55（2.74-7.56） | ***P* ＜ 0.001** |

Model1: Crude model;

Model2: Adjusted for sex, age, smoking, history of hypertension, diabetes;

Model3: Adjusted for sex, age, smoking, history of hypertension, diabetes, SBP, anti-hypertension medication, antidiabetic agents, lipid-lowering medication, FBG, TC.
